# Quantitative proteomics-based analyses performed on pre-eclampsia samples in the 2004–2020 period: a systematic review

**DOI:** 10.1186/s12014-021-09313-1

**Published:** 2021-01-26

**Authors:** Rosana Navajas, Fernando Corrales, Alberto Paradela

**Affiliations:** Functional Proteomics Facility, Centro Nacional de Biotecnología (CNB-CSIC), ProteoRed-ISCIII, Madrid, Spain

**Keywords:** Proteome, Proteomics, Biomarkers, Pre-eclampsia, Pregnancy, Plasma, Placenta, Urine, Cohort studies

## Abstract

**Background:**

Quantitative proteomics is an invaluable tool in biomedicine for the massive comparative analysis of protein component of complex biological samples. In the last two decades, this technique has been used to describe proteins potentially involved in the pathophysiological mechanisms of preeclampsia as well as to identify protein biomarkers that could be used with diagnostic/prognostic purposes in pre-eclampsia.

**Results:**

We have done a systematic review of all proteomics-based papers describing differentially expressed proteins in this disease. Searching Pubmed with the terms pre-eclampsia and proteomics, restricted to the Title/Abstract and to MeSH fields, and following manual curation of the original list, retrieved 69 original articles corresponding to the 2004–2020 period. We have only considered those results based on quantitative, unbiased proteomics studies conducted in a controlled manner on a cohort of control and pre-eclamptic individuals. The sources of biological material used were serum/plasma (n = 32), placenta (n = 23), urine (n = 9), cerebrospinal fluid (n = 2), amniotic fluid (n = 2) and decidual tissue (n = 1). Overall results were filtered based on two complementary criteria. First, we have only accounted all those proteins described in at least two (urine), three (placenta) and four (serum/plasma) independent studies. Secondly, we considered the consistency of the quantitative data, that is, inter-study agreement in the protein abundance control/pre-eclamptic ratio. The total number of differential proteins in serum/plasma (n = 559), placenta (n = 912), urine (n = 132) and other sources of biological material (n = 26), reached 1631 proteins. Data were highly complementary among studies, resulting from differences on biological sources, sampling strategies, patient stratification, quantitative proteomic analysis methods and statistical data analysis. Therefore, stringent filtering was applied to end up with a cluster of 18, 29 and 16 proteins consistently regulated in pre-eclampsia in placenta, serum/plasma and urine, respectively. The systematic collection, standardization and evaluation of the results, using diverse filtering criteria, provided a panel of 63 proteins whose levels are consistently modified in the context of pre-eclampsia.

## Background

Pre-eclampsia is a multifactorial disease that affects 5–10% of pregnancies and is one of the leading causes of fetal and maternal morbidity and mortality, accounting for 40% of fetal deaths worldwide [1]. Diagnosis is established after detecting symptoms of new onset hypertension (systolic blood pressure > 140 mm Hg, diastolic blood pressure > 90 mm Hg), proteinuria (> 0.3 g in 24 h collected urine sample) and swelling. Symptoms typically appear between weeks 20–34 (early-onset) or later (late onset). Pre-eclampsia has a wide spectrum with regard to presentation, time of onset, and severity. Women may develop intense headaches or visual changes, acute renal failure, epigastric pain, liver injury, pulmonary edema, thrombocytopenia and hemolysis. In the most severe cases (eclampsia) seizures might appear [2]. Furthermore, pre-eclampsia may affect both women and children later in life [3]. Women are prone to develop diabetes, hypertension, coronary artery disease and stroke. Children present increased risk for metabolic and cardiovascular disease [4, 5].

Past research has led to important advances in understanding the pathogenesis of pre-eclampsia. However, although several hypotheses exist on the main triggering factors, there is no consensus on the etiology of this disease and neither is there preventive strategy [2]. Most models point to a dysregulation of the angiogenic balance that modulates the early placental vascular development and trophoblast invasion. Vascular endothelial growth factor (VEGF) and the homologous Placental growth factor (PlGF), expressed at high levels by human placenta, are essential for embryonic vasculogenesis (formation of new blood vessels) and angiogenesis (blood vessels branch to form new blood vessels) [6, 7]. Both pro-angiogenic factors bind to VEGFR1 (Flt-1) receptor, a signaling protein involved in both vasculogenesis and angiogenesis. A soluble form of this receptor, sFlt-1, has been described as released by the placenta into the maternal circulation. Interestingly, sFlt-1 levels have been found to be upregulated in women with pre-eclampsia [8]. sFlt-1 would bind both VEGF and PlGF, preventing their binding to the membrane-bound receptor Flt-1, and causing soluble VEGF/PlGF levels to decrease. Another antiangiogenic protein found increased in samples obtained from women with pre-eclampsia is soluble Endoglin (sEng) [9]. sEng inhibits the interaction of another angiogenic factor, TGF-β1, to its receptor, further contributing to the alteration of the angiogenic balance [10]. These important findings have made it possible the use of these proteins as diagnostic biomarkers of the disease [11]. Calculation of the sFlt-1/PlGF ratio is currently being used as a measurement that reflects the alteration of the angiogenic balance [12]. Interestingly, the ratio increases before clinical symptoms start and thus may help in predicting pre-eclampsia. However, although sFlt-1/PlGF ratio is potentially useful, studies performed to date disagree about cutoff values, gestational age for screening and several other important parameters [13]. Other studies point to the relationship of pre-eclampsia with endoplasmic reticulum stress syndrome, causative of toxic fibrillar deposits found in pre-eclamptic placenta tissues. Consequently, misfolded and aggregated proteins would lead to an imbalance of angiogenic factors and poor vascularization [14, 15].

This lack of consensus on the main causes of the disease fuel the search for new biomarkers for either diagnosis or prognosis. According to NIH Biomarker definitions working group, a biomarker is “a characteristic that is objectively measured and evaluated as an indicator of normal biological processes, pathogenic processes, or pharmacologic responses to a therapeutic intervention” [16]. Although the definition of biomarker encompasses different parameters, the term commonly refers to molecules of biological origin. Its importance in biology and biomedicine is undeniable: a search of the term Biomarker limited to the article title, yielded more than 26,100 results (PubMed, March 2020). In the case of pre-eclampsia, understanding its etiology and the aim of predicting the onset of the disease before detecting the first clinical symptoms has fueled numerous studies. Due to the complexity of this pathology in terms of maternal predisposition, genetic inheritance and association of several medical conditions (diabetes mellitus, chronic hypertension, metabolic syndrome and others) with increased pre-eclampsia risk, omics technologies have become the ideal tools to find new biomarkers. These should help to explain the molecular mechanisms implicated and, potentially, could be used with diagnostic/prognostic purposes [17]. Omics technologies focus in the massive and unbiased detection and quantification of genes (genomics), mRNA (transcriptomics), proteins (proteomics) and metabolites (metabolomics), taking advantage of methods such as high-resolution liquid chromatography coupled to mass spectroscopy (LC–MS), next generation sequencing (NGS), and chip arrays [18]. By using omics platforms to compare control and pre-eclampsia samples, a plethora of hallmarks have been reported at the level of mRNA [19, 20], metabolites [21, 22], DNA methylation sites [23], miRNAs [24],proteins and PTM [25], associated with the disease.

Proteomics, both for the identification and quantification of proteins and the determination of their post-translational modifications, has progressed enormously in recent decades [26]. Two-dimensional gel electrophoresis (2D-PAGE) followed by the identification of the spots of interest using MALDI-TOF mass spectrometry [27], have been replaced by high-performance liquid chromatography coupled to high-resolution mass spectrometry (LC–MS). The latest state-of-the-art LC–MS platforms allow for the identification and quantification of thousands of proteins in just 1–2 h. Besides, the use of labeling reagents that allow the simultaneous analysis of 6, 8, 11 and up to 16 samples [28], facilitates the comparative analysis of tens of control and problem samples in short periods (e.g. one day). In addition to these improvements, new and better experimental protocols have been developed for obtaining, storing and processing samples, as well as experimental approaches to enrich certain molecular characteristics such as phosphorylation, methylation or acetylation [29, 30]. For these reasons, proteomics is an indispensable and invaluable tool in biomedicine for approaching phenotypes by the massive comparative analysis of protein component of complex biological samples. However, it is essential to be aware that although proteomics has already reached maturity, it has experienced a continuous technical evolution during the last 20 years and therefore systematic and careful analyses of data in the literature are needed to reach unified outcomes on specific topics.

We systematically analyzed the proteomics-based results published from 2004 to 2020 about pre-eclampsia (Additional file 1: Figure S1). The terms “pre-eclampsia AND proteomics” were used for the PubMed search, retrieving 60 articles when search was restricted to the Title/Abstract field. A second and independent search, restricted to MeSH terms returned 90 studies. We removed non-English studies, reviews and duplicates found between both lists. Finally, a manual evaluation of the remaining studies was performed. We only considered quantitative proteomic studies that aimed at systematic and unbiased comparison of human samples from control individuals and patients diagnosed with pre-eclampsia. Taken all these restrictions together, the total number of original studies dropped to 69. The source of biological material is variable although serum/plasma, placenta and, to a lesser extent, urine, predominates. We have also collected other minor sources of biological material (e.g., cerebrospinal fluid) although for practical reasons they are grouped into a single section. The number of control/pre-eclampsia samples compared in the studies varies from very few to tens of samples. There were also large differences in terms of sample collection methods, patient stratification (severe/mild, early/late onset, pre-term/term, with and without Intrauterine growth restriction), and statistical analysis of quantitative data. Protein names as described in the different studies have been unified, when possible, to UniprotKB Protein Accession and gene names, while maintaining the protein name as described by the authors. In those cases where a UniprotKB equivalent could not be found, results were not considered. We have divided the results in chapters according to the source of biological material. To our knowledge, this is the largest compilation and classification of quantitative proteomic data related to pre-eclampsia. With the aim of facilitating the understanding of the results, most relevant data are shown in the form of summary tables, while in supplementary materials more detailed information can be found.

## Placenta

Dysfunctions associated with the placenta are at the heart of many pathologies related to pregnancy, including pre-eclampsia [31]. Indeed, the only effective available treatment that suppresses pre-eclampsia associated symptoms is the removal of the placenta through induction of labor. Experimental evidence suggests that poor remodeling of maternal spiral arteries due to a shallow trophoblast (of placental origin) invasion and abnormal placental formation in early pregnancy is the main causative agent of pre-eclampsia [2]. Continuous interplay between placental and maternal tissues is reflected in the synthesis of numerous factors, both of placental and maternal origin [32]. Detection and quantification of these changes by massive analysis techniques such as proteomics, especially at early stages, can help to identify mechanisms involved in the origin and development of the disease. In addition, they can act as prognostic and diagnostic biomarkers. Although placenta is a promising source of potential biomarkers, accessing placental cells or tissues is not easy, particularly at early stages due to practical and ethical reasons. Sampling by invasive methods, such as amniocentesis or chorionic villus, represents a significant risk for the mother and the fetus and limits the potential clinical use of the candidate biomarkers [33].

Despite all these major drawbacks, samples of placental origin obtained at different developmental stages and severity conditions have been used in quantitative studies for the assessment of predictive markers associated with pre-eclampsia. Since the first data published in 2007, we have found a total of 23 publications focused on the proteomic analysis of placentas obtained from either pre-eclamptic or control individuals (Additional file 2: Table S1). All samples were collected at term, either after natural delivery or after cesarean section. No significant differences were found regarding the gestational age at which the samples were obtained, although in most cases the pre-eclamptic placental samples were obtained 2–4 weeks earlier than the control samples. Some studies distinguished between early and late onset pre-eclampsia, or mild and severe pre-eclampsia, while others did not. Differences have also been found in the number of samples analyzed, ranging from a few individuals to a maximum of 25 control and 25 test samples. Most studies used primary placental tissue, but some used cytotrophoblast primary cultures as a source of biological material. Regarding the type of technology used, published works have followed the evolution of proteomic technologies in the last two decades. Thus, first publications used 2D-PAGE approaches followed by identification by MALDI-TOF mass spectrometry, but this technology has been mostly substituted by LC–MS-based quantitative strategies in their different versions (DDA label-free, isobaric labeling, DIA). Taken together, these variations could explain the high data-dispersion found. Considering all data, 1200 differentially regulated proteins with statistical significance are described in the 23 references considered, corresponding to 913 non-redundant proteins. However, most of these 913 putative differentially regulated proteins have only been defined as such in a single study, and they have been excluded for subsequent analysis. Thus, only 154 (16.9%) proteins are described in 2 or more works, 46 (5%) in 3 or more, and finally, only 16 (1.8%) proteins have been defined as potentially related to the pathology in 4 or more studies. The complementarity across studies, likely arises from the diversity of proteomic approaches used, sample collection methods and the nature of human samples (phenotypic and genotypic variation).

Focusing on those proteins most frequently detected, we have also found differences in the direction of change (Table 1). Thus, among the 16 proteins identified in 4 or more works, only CLIC3, ALB and HSPB1 show consistent case/control ratios. In contrast, 6 proteins (FN1, ANXA1, ANXA2, HSPA8, CANX, PDIA3) showed an erratic pattern, contradictory protein expression levels and poor correlation between studies. Finally, another set of proteins (PLG, FGG, CAT, CYP11A1, ANXAA4, HSPA5, PRDX2) show a less consistent pattern than the first group, but clearly point to a main trend. Finally, within the set of proteins described in 3 publications, we rescued those with consistent trends. There are 8 proteins (FGB, GAPDH, HBZ, ANXA6, FLT1, ATIC, ACTG1 and PAPPA2) that complete the set of 18 proteins with a robust up or down regulation in control and pre-eclamptic samples.

**Table 1:** Full list of the 18 proteins whose expression patterns change consistently when comparing pre-eclamptic versus control placenta samples

Accesion^a^	Gene name^a^	Protein name^a^	PE > C^b,d^	PE < C^c,d^
O95833	CLIC3	Chloride intracellular channel protein 3	[52–56]	
P00747	PLG	Plasminogen	[53, 57, 58]	[52]
P02008	HBZ	Hemoglobin subunit zeta	[52, 53, 59]	
P02675	FGB	Fibrinogen beta chain		[57, 59, 60]
P02679	FGG	Fibrinogen gamma chain	[60]	[52, 57, 59]
P02768	ALB	Albumin		[52–54, 57, 60]
P04040	CAT	Catalase	[57, 59, 61, 62]	[52, 53]
P04406	GAPDH	Glyceraldehyde-3-phosphate dehydrogenase	[53, 62, 63]	
P04792	HSPB1	Heat shock protein beta-1	[54, 56, 60, 62, 63]	
P05108	CYP11A1	Cholesterol side-chain cleavage enzyme, mitochondrial	[52, 53, 64]	[57]
P08133	ANXA6	Annexin A6	[53, 57, 58]	
P09525	ANXA4	Annexin A4	[60, 63, 65]	[57]
P11021	HSPA5	Endoplasmic reticulum chaperone BiP	[54, 56, 57, 63]	[61, 62]
P17948	FLT1	Vascular endothelial growth factor receptor 1	[52, 57, 65]	
P31939	ATIC	Bifunctional purine biosynthesis protein ATIC	[52, 53, 61]	
P32119	PRDX2	Peroxiredoxin-2	[54, 57, 59, 61]	[55, 66]
P63261	ACTG1	Actin, cytoplasmic 2		[55, 60, 67]
Q9BXP8	PAPPA2	Pappalysin-2	[52, 53, 65]	

^a^According to UniprotKB

^b^PE > C: expression levels have been described to be higher in pre-eclampsia versus control samples

^c^PE < C: expression levels have been described to be lower in pre-eclampsia versus control samples

^d^Numbers correspond to bibliographic references.

## Plasma/serum

Quantitative proteomic analysis of plasma or serum samples with the aim of finding disease-associated biomarkers has long been a pivotal goal of the academic community. There are several and justified reasons for this: on the one hand, it is an easily accessible source of biological material that can be obtained at different time points of a physiological or pathological process; second, blood circulation ensures the presence of proteins secreted by all tissues including placental or decidual tissues. Finally, plasma/serum samples remain reasonably stable under standard conditions of conservation [34]. Nevertheless, there are also some disadvantages, among which the complexity and dynamic range of serum/plasma proteome are the most important. Dynamic range of concentration expands more than 10 logs and is the highest found in a biological sample. This is due to the presence of a small set (n $\sim$ 20) of extraordinarily abundant proteins (i.e., albumin, antibodies, apolipoproteins, complement proteins and others), which represent 99% of the protein mass in plasma/serum and make difficult the identification and quantification of the less-abundant proteins [35]. Therefore, detection of disease-associated biomarkers that often are commonly low-abundant proteins, often require previous sample immunodepletion to get rid of the top abundant proteins. Other alternatives, such as sample prefractionation, have been employed. A second negative aspect is that the aforementioned ability of serum/plasma to collect molecular species throughout the body implies that proteins specifically secreted by the tissue of interest (i.e., placental tissue) might be diluted to undetectable levels.

Despite these pros and cons, quantitative proteomic serum/plasma analysis has attracted the interest of the medical and academic community over the past two decades. The number of published articles describing quantitative proteomic results using plasma and serum pre-eclamptic samples is 32. We have combined published results using serum and plasma for their obvious functional and compositional similarities, although the use of plasma samples prevails over that of serum. First published references found date back to 2004 and, as mentioned above, proteomic technologies used are very diverse, with a clear predominance of 2D-PAGE followed by MALDI-TOF MS during the first period, which are progressively replaced by LC-MSMS-based techniques. Antibody-based specific depletion of highly-abundant proteins was commonly used, but other techniques aiming to enrich specific protein subsets, such as COFRADIC [36] or methods to reduce the dynamic concentration range in the sample [37, 38], were also employed. As sampling was done at different timing points, we have classified the studies into trimesters, according to when the sample was taken (first, second or third, respectively), with most cases collected along the second and third trimester and some cases at term (Additional file 3: Table S2). This aspect makes an important difference with placenta, where almost all samples were obtained at term. Finally, we have also found differences in sample classification according to disease severity (mild, severe) or to the onset (early, late) of the pathology, as well as in the number of samples analyzed, ranging from a few individuals to a maximum of 90 and 76 control and test samples, respectively. All the aforementioned variables explain, at least partially, the dispersion of the quantitative proteomic data.

We have found 32 published references, describing a total of 890 differentially regulated proteins with statistical significance in the context of the pathology. These proteins correspond to 559 non-redundant proteins. However, a significant percentage of these putative differentially regulated proteins has only been defined in a single study and, therefore, were excluded for subsequent analysis. Overall, 185 (33.1%) proteins are reported in 2 or more works, 97 (17.4%) in 3 or more, and finally, only 60 (10.7%) proteins have been defined as potentially related to the pathology in four or more studies. We also have found that quantitative values for a significant proportion of proteins are widely dispersed (Additional file 3: Table S2), but 29 proteins reported in four or more data sets show consistent variation trends across studies. These data clearly increase our current knowledge of the molecular pathways associated with pre-eclampsia. For example, one of the proteins found to be consistently overexpressed in pre-eclampsia is Endoglin (Table 2), whose soluble form (sEndoglin) was one of the first protein biomarkers described as associated with pre-eclampsia [9]. Another protein of interest is the Placental growth factor, whose levels, in line with previous studies, are decreased in plasma as it is scavenged by sFlt1 protein. Taken together, this relatively small list of circulating proteins represents a promising basis for the design of analytical tools, along with other biomarkers previously described, for a more precise follow-up of pre-eclamptic patients.

**Table 2:** Full list of the 29 proteins whose expression patterns change consistently when comparing pre-eclamptic versus control serum/plasma samples

Accesion^a^	Gene name^a^	Protein name^a^	PE > C^b,d^	PE < C^c,d^
P00740	F9	Coagulation factor IX	[68]	[69, 70]
P00751	CFB	Complement factor B	[71]	[48, 70, 72]
P01009	SERPINA1	Alpha-1-antitrypsin	[48, 73–75]	[71]
P01023	A2M	Alpha-2-macroglobulin		[68, 71, 74, 76, 77]
P02649	APOE	Apolipoprotein E	[74, 75, 78]	[76]
P02679	FGG	Fibrinogen gamma chain	[68, 74, 78, 79]	
P02741	CRP	C-reactive protein	[68, 69, 75, 76]	[80]
P02746	C1QB	Complement C1q subcomponent subunit B	[76]	[68, 69, 72]
P02751	FN1	Fibronectin	[69–71, 74, 75, 79, 81]	[77]
P02760	AMBP	Protein AMBP	[73–76]	[75]
P02766	TTR	Transthyretin		[14, 74, 81, 82]
P02787	TF	Serotransferrin	[48, 69, 78, 79]	
P04114	APOB	Apolipoprotein B-100	[77]	[69, 74, 81]
P04196	HRG	Histidine-rich glycoprotein	[68, 69, 75, 82]	[72]
P05155	SERPING1	Plasma protease C1 inhibitor	[71]	[69, 70, 77]
P08514	ITGA2B	Integrin alpha-IIb	[70, 83]	
P08697	SERPINF2	Alpha-2-antiplasmin	[84]	[69, 70, 76]
P09237	MMP7	Matrilysin	[70, 83]	
P0C0L5	C4B	Complement C4-B		[68, 77, 81, 83]
P10909	CLU	Clusterin	[68, 73–75, 77–79, 81, 85, 86]	[76]
P11464	PSG1	Pregnancy-specific B-1 glycoprotein 1	[77]	[69, 75, 76, 84]
P17813	ENG	Endoglin	[69, 77, 80, 87, 88]	
P20742	PZP	Pregnancy zone protein	[68, 69]	[69, 74, 75]
P22352	GPX3	Glutathione peroxidase 3	[89]	[74–76]
P36955	SERPINF1	Pigment epithelium-derived factor	[71, 72, 74, 81]	[76]
P49763	PGF	Placenta growth factor		[70, 83, 87]
Q14624	ITIH4	Inter-alpha-trypsin inhibitor heavy chain H4	[68, 75, 76]	[72]
Q15485	FCN2	Ficolin-2		[70, 80, 90]
Q16610	ECM1	Extracellular matrix protein 1	[87]	[69, 76, 77]

Reference 69 reports two independent data for F9, C1QB, FN1, TF, APOB, HRG, SERPING1, SERPINF2, PSG1, ENG, PZP and ECM1 and four independent data for CRP; reference 70 reports two independent data for FCN2, CFB, MMP7 and PGF and three independent data for ITGA2B; reference 83 reports two independent data for MMP7.

^a^According to UniprotKB

^b^PE > C: expression levels have been described to be higher in pre-eclampsia versus control samples

^c^PE < C: expression levels have been described to be lower in pre-eclampsia versus control samples

^d^Numbers correspond to bibliographic references

## Urine

In principle, urine is an excellent sample source for proteomic studies since its collection is easier than that of blood and can be performed at different time points along the progression of the disease with minimal risk for the patient. Urine contains plasma proteins that pass through the glomerular filtration barrier [39] and might indicate the physiological or pathological condition of the organism. The urine-associated proteome is less complex than that of serum or plasma, which depending on the specific circumstances may or may not be an advantage. Under non-pathological conditions, around 30% of urinary proteins derive from circulating peptides and small proteins present in blood and secreted into the urine. The remaining 70% originates from the kidney and urinary tract [33]. Therefore, urine proteomics can be used to detect early physiological and pathological changes occurring in the organism. However, it should be kept in mind that one of the most characteristic symptoms of pre-eclampsia is proteinuria, which reaches 300 mg of protein or greater in a 24-h urine specimen [2]. This enormous increase in the amount of protein in urine is a consequence, and not a cause, of the pathology and may be misleading in regard to the molecular mechanisms associated with the disease. Standardization during sample-collection is important to reduce the impact of sample variations in urine protein concentration due to fluid intake or exercise.

Despite the great interest of urine as a source of possible protein biomarkers, the number of articles published so far is relatively small. We found only 9 publications, covering the period from 2008 to present. As in the previous cases, there is a wide spectrum in terms of the proteomic technologies used. These include the use of SELDI-TOF MS, a type of mass spectrometry that achieved some popularity in the early years of this century but has been discontinued due to its limited reproducibility, resolution and low sensitivity. Additionally, there are huge differences regarding the number of samples analyzed, ranging from a few tens to a few hundreds. Likewise, there are significant differences in the clinical stratification of the samples, which includes results corresponding to early, late, mild and severe pre-eclampsia, along with others that do not distinguish these categories. These differences explain, at least partially, the high data dispersion observed.

In the nine publications considered, 167 different proteins that correspond to 132 non-redundant proteins are described as differentially regulated with statistical significance (Additional file 4: Table S3). Most of these proteins are described in a single study (80%), so considering those reported in at least two studies, 24 (18.2%) and 6 (1.9%) proteins have been identified and quantified in at least 2 or 3 works, respectively. Only 2 proteins, SERPINA1 and ALB, are described in 4 or more works. From the set of 24 proteins, 8 (33.3%) showed inconsistent trends across studies while 16 (66.6%) were similarly up-or down-regulated in all cases (Table 3). Although this heterogeneity likely results from the above-mentioned variables, the presence of proteins like SERPINA1 clearly reflect underlying physio-pathological processes.

**Table 3:** Full list of the 16 proteins whose expression patterns change consistently when comparing pre-eclamptic versus control urine samples

Accesion^a^	Gene name^a^	Protein name^a^	PE > C^b,d^	PE < C^c,d^
P00450	CP	Ceruloplasmin	[91–93]	
P00747	PLG	Plasminogen	[93, 94]	
P01009	SERPINA1	Alpha-1-antitrypsin	[91–97]	
P01024	C3	Complement C3	[91, 93]	
P01042	KNG1	Kininogen-1		[92, 93]
P02452	COL1A1	Collagen alpha-1(I) chain	[96, 98]	
P02461	COL3A1	Collagen alpha-1(III) chain	[96, 98]	
P02768	ALB	Albumin	[92, 93, 96, 97]	
P02787	TF	Serotransferrin	[93, 94]	
P05154	SERPINA5	Plasma serine protease inhibitor		[93, 94]
P06396	GSN	Gelsolin		[92, 93]
P07911	UMOD	Uromodulin	[96, 98]	
P41222	PTGDS	Prostaglandin-H2 D-isomerase		[92, 93]
P68871	HBB	Hemoglobin subunit beta	[93, 96]	
P98160	HSPG2	Basement membrane specific eparin sulfate proteoglycan core protein		[92, 93]
Q12907	LMAN2	Vesicular integral-membrane protein VIP36		[92, 93]

^a^According to UniprotKB

^b^PE > C: expression levels have been described to be higher in pre-eclampsia versus control samples

^c^PE < C: expression levels have been described to be lower in pre-eclampsia versus control samples

^d^Numbers correspond to bibliographic references.

Reference 92 reports two independent data for KNG1, CP, SERPINA1, ALB, GSN, PTGDS, HSPG2 and LMAN2; reference 96 reports two independent data for SERPINA1, COL1A1, COL3A1, ALB, UMOD and HBB.

## Other sources

Finally, in this section we have collected those experimental contributions made through the quantitative proteomic analysis of samples obtained from other sources of biological material, such as cerebrospinal fluid (CSF) [40, 41], amniotic fluid [42, 43], and decidual tissue [44]. The arguments used to rationalize the use of these sources are relevant, ranging from the suitability of the cerebrospinal fluid as a source of potential markers associated with the neurological manifestations characteristic of the most severe cases of pre-eclampsia, to the physical proximity of the amniotic fluid with both the fetus and other pregnancy-related tissues. Decidua was chosen in the unique reference available to date due to its critical role in shaping the immune microenvironment between the mother and the fetus. The limited number of published articles (n = 5) available makes it very difficult to select those protein biomarkers consistently detected and quantitated in an independent way. Therefore, we will only emphasize here putative protein biomarkers that confirm those included in the previous tables or that are consolidated references in the field (Additional file 5: Table S4). HBB protein appears both in decidual tissue and in urine. In both cases, this protein is upregulated in pre-eclamptic samples compared to controls. A similar situation occurs in the case of SERPINA1, which has been described as overexpressed in pre-eclampsia either in decidua or in serum/plasma and urine samples. In contrast, both TTR and FGB, reported as negatively regulated in pre-eclampsia compared to control samples in serum/plasma and placental tissue, respectively, showed an opposite regulation in amniotic and decidual tissue. These selected examples illustrate that one of the challenges ahead of molecular data interpretation and translation into the clinical scenario is to discover the links between the observations in different tissues to obtain a description at the organism level.

## Discussion

In this article, we have systematically reviewed all the available bibliographic references reporting data from quantitative proteomics-based analyses of control and pre-eclamptic samples. Our search rendered a list of 69 articles published in the 2004–2020 period. We have only considered those results based on quantitative, unbiased proteomics studies conducted in a controlled manner on a cohort of control and pre-eclamptic individuals. Most of the published works have been carried out using serum/plasma (n = 32) and placenta (n = 23) as sources of biological material, although there is also a significant number of contributions using urine (n = 9). Finally, a small number of publications used cerebrospinal fluid (n = 2), amniotic fluid (n = 2) and decidual tissue (n = 1).

It is worth noting that there are wide differences in the number of samples used in each study, ranging from a few samples to a few hundred. The differences are also significant in relation to the period in which the samples were obtained as well as to the degree of precision in the classification of the disease. Regarding the first aspect, in some studies, mainly those focused on the analysis of samples of placental origin, samples were collected at the end of pregnancy, while analysis of serum/plasma and urine samples enables the collection of samples throughout the pregnancy and therefore, a more precise monitoring of changes at the proteome level. Regarding the second aspect, some studies are limited to the comparison of samples simply defined as control/pre-eclamptic, while other studies accomplished a much more accurate stratification of patients, identifying early and late variants of the disease, or distinguishing the most severe cases from those less aggressive. The experimental approaches used faithfully reflect the technological evolution in the proteomics field. Thus, in the first years 2D-PAGE separation followed by MALDI-TOF-TOF mass spectrometry analysis was the standard technique. Over time, this technique has been almost completely substituted by LC–MS/MS-based approaches, either in combination with isobaric chemical labeling (i.e., iTRAQ or TMT) or using label-free techniques. In the last years, the use of Data Independent Acquisition (DIA) analysis is also becoming relevant (Additional file 6: Table S5).

The experimental variability, added to the natural variation of human samples, is the most likely cause of the large number of proteins published as differentially regulated in pre-eclampsia. Thus, the total sum of proteins defined as potential biomarkers in serum/plasma (n = 559 unique proteins), placenta (n = 913), urine (n = 132) and other sources of biological material (n = 26), reaches 1630 proteins. Nevertheless, this number drops to 1411 non-redundant proteins. This figure, which represents roughly 7% of the human proteome, is unexpectedly high and, therefore, demands a critical evaluation to discriminate useful information from what might be considered as experimental noise.

With this goal, we filtered the overall results based on two complementary criteria. On the one hand, we have only taken into account those proteins described in at least two (urine), three (placenta) and four (serum/plasma) articles. Due to the small number of studies carried out on CSF, amniotic fluid and decidual tissue, this criterion could not be applied in these samples. Secondly, we considered the consistency of the quantitative data between control and pre-eclamptic samples. Proteins showing inconsistent results between studies were systematically rejected (for a more exhaustive description of the methodology used, see Additional file 7: Supplementary methods). As expected, the application of these strict criteria greatly reduced the initial number of differentially regulated proteins and, therefore, of potential biomarkers associated with pre-eclampsia. Thus, the initial number of proteins described in placenta, serum/plasma and urine sources, collapsed to 18, 29 and 16, respectively, corresponding to those proteins described in two or more independent studies, using unrelated cohorts of control and pre-eclamptic samples and different experimental approaches, and showing similar quantitative changes in the context of the pathology. This is, therefore, a consolidated set of results, of great interest to the clinical and academic community. It is noteworthy that proteins shown in Tables 1, 2, 3 are described in 47 of the 69 articles used for this compilation. In other words, quantitative data published in approximately 1/3 of the proteomic analyzes performed in the 2004–2020 period were inconsistent or were not confirmed in similar studies.

The overlap between the different sets of proteins is very low. There is no protein common to all three sets, whereas FGG (Fibrinogen gamma chain) is shared between placenta and serum/plasma, PLG (plasminogen) and ALB (albumin) between placenta and urine and, finally, SERPINA1 (alpha-1-antitrypsin) and TF (transferrin) are common to urine and serum/plasma studies. SERPINA1 was also detected in decidual tissue. Interestingly, the direction of change in the expression levels of some proteins depends on the tissue or biological fluid considered. For example, in serum/plasma, FGG protein is more abundant in pre-eclampsia versus control samples, but in placental tissue the situation is exactly the opposite. This may be due, among other reasons, to a continuous release of protein from the placenta to the plasma. On the other hand, well-known pre-eclampsia-associated biomarkers are present in our data sets providing reliability to the results. As an example, the increased levels in pre-eclamptic serum/plasma observed for endoglin, accompanied by decreasing Placental growth factor levels, have been previously described [9] and points to an imbalance in the angiogenic process proposed as one of the disease triggering factors. This imbalance is also affected by the increased levels of Vascular endothelial growth factor receptor 1 in pre-eclamptic placentas (Table 1), probably related with an increase in serum/plasma of the soluble form (sFlt1) of this receptor.

Oxidative stress is thought to have a key role in normal and defective placental development. In pre-eclampsia, an imbalance seems to exist between antioxidant and pro-oxidant mechanisms. Defective spiral artery remodeling detected in affected pregnancies seems to be the major cause of this alteration [45]. We show here that the levels of various proteins related to oxidative stress change consistently in pre-eclampsia. Catalase (CAT) and peroxiredoxin-2 (PRDX2), both involved in protecting cells from the toxic effects of hydrogen peroxide and organic hydroperoxides, are more abundant in pre-eclamptic than in control placentas. Interestingly, an enzyme with similar function such as glutathione peroxidase-3 (GPX3) has been described as downregulated in pre-eclamptic plasma samples.

The complement system plays an essential role in the innate immunity and inflammation, providing a link between innate and adaptive immunity. Several studies have suggested an association between inappropriate or excessive activation of the complement system at the placenta and pre-eclampsia[46]. A significant percentage of the more than 30 proteins that are part of the complement system have been detected in the studies collected here, particularly from plasma samples, although quantitative data are contradictory in many cases (Additional file 3: Table S2). We have detected consistent changes for Complement factor B (CFB), complement C1q subcomponent subunit B (C1QB) and Complement C4B (C4B). In all cases, expression levels decreased in pre-eclamptic plasma samples. Other proteins involved in the complement system, such as Plasma protease C1 inhibitor (SERPING1) or Ficolin-2 (FCN2) appear also to be downregulated. On the contrary, analysis of urine samples shows an increased presence of complement C3 (C3) in pre-eclampsia.

On the other hand, it is well known that the accumulation of misfolded proteins is at the origin of multiple pathologies, including pre-eclampsia [47]. In this context, it is not surprising to find deregulated levels of proteins involved in protein folding and stabilization, maintenance of protein homeostasis, as well as in the inhibition of the aggregation of misfolded proteins. These include Heat shock protein beta-1 (HSPB1) and Endoplasmic reticulum chaperone BiP (HSPA5), both found upregulated in pre-eclamptic placental tissues, while Alpha-2-macroglobulin (A2M) and Pregnancy zone protein (PZP) have been described as downregulated in pre-eclamptic plasma. It has been suggested that PZP deficiency could contribute to the accumulation of misfolded proteins in pre-eclampsia [15].

Despite the complementarity observed across the quantitative proteomic studies focused on pre-eclampsia analyzed here, that result from uncontrolled variables like the use of different technologies, number of samples, statistical processing of data and classification and stratification of the samples, the filtering criteria used allowed us to extract useful quantitative proteomic information. The difficulty in collecting appropriate samples reflecting the entire spectrum of the disease is a major and essential issue to identify predictive biomarkers for the early detection of women at risk [48]. The refined list of proteins we offer here represents a good starting point to perform further validation experiments (e.g., western blot or targeted proteomics) on a large cohort of samples. Particularly interesting for this purpose is the SRM/MRM targeted proteomic analysis, which makes it possible the specific monitoring of large sets of proteins in a single assay. On-line tools, such as SRMAtlas (http://www.srmatlas.org/) greatly facilitate the design of SRM / MRM methods. This tool has been used to raise a Additional file 8: Table S6 showing the proteotypic peptides and transitions corresponding to the proteins in Tables 1, 2, 3, needed to perform SRM-based targeted proteomics experiments. Finally, we would like to insist in the importance of robustness and reproducibility in sample preparation for subsequent proteomic analysis [49–51].

## Conclusion

Proteomics has now reached maturity upon integration of diverse tools. The latest LC–MS platforms enable the identification and quantification of thousands of proteins with great accuracy and reproducibility. This, combined with standardized collection and automated sample treatment, will lead in the short term to more robust proteomic quantitative data, and biomarker identification in all kinds of biological issues, particularly in clinical studies.

## Supplementary Information

**Additional file 1: Figure S1.** Summary of the selection and filtering process of the scientific literature used in this review.

**Additional file 2: Table S1.** Complete list of proteins described in the literature as differentially regulated from placental samples.

**Additional file 3: Table S2.** Complete list of proteins described in the literature as differentially regulated from serum/plasma samples.

**Additional file 4: Table S3.** Complete list of proteins described in the literature as differentially regulated from urine samples.

**Additional file 5: Table S4.** Complete list of proteins described in the literature as differentially regulated from other sample sources (cerebrospinal fluid, amniotic fluid, decidual tissue).

**Additional file 6: Table S5.** Summary of the experimental techniques used in the scientific literature cited in this review.

**Additional file 7.** Supplementary methods: acceptance criteria for the proteins appearing in Tables 1, 2, 3.

**Additional file 8: Table S6.** Table summarizing the peptides and transitions for SRM validation of proteins of Tables 1, 2, 3.

## Data Availability

The data used in the preparation of this manuscript have been obtained, processed and published by other authors. These are accessible through the publications whose references appear at the end of the manuscript. Throughout the process of obtaining the data for writing this manuscript, we have encountered different situations, from published works that facilitated access to all the data used (including raw data) to some publications that did not include said data or included them only partially. In these latter cases, we have contacted the authors directly in order to obtain the complete list of data, with different degrees of response to our requirements. In those cases where we did not receive a response from the authors, we have decided not to include their data in this review.

## Abbreviations

CSF: Cerebrospinal fluid
DDA: Data-dependent acquisition
DIA: Data-independent acquisition
2D-PAGE: Two-dimensional polyacrylamide gel electrophoresis
LC–MS: Liquid chromatography coupled to high-resolution mass spectrometry
MALDI-TOF MS: Matrix-Assisted Laser Desorption/Ionization mass spectrometry
MeSH: Medical Subject Headings
PlGF: Placental growth factor
PTM: Posttranslational modifications
sEng: Soluble Endoglin
VEGF: Vascular endothelial growth factor
VEGFR1: Vascular endothelial growth factor receptor 1
