# Supplementary material for: Quantitative proteomics-based analyses performed on pre-eclampsia samples in the 2004–2020 period: a systematic review
Source: Clin Proteomics. 2021 Jan 26;18:6. doi: 10.1186/s12014-021-09313-1 (PMC7836571; doi:10.1186/s12014-021-09313-1)
Supplement: Supplementary file 1 — Additional file 1: Figure S1. Summary of the selection and filtering process of the scientific literature used in this review. [file 12014_2021_9313_MOESM1_ESM.pptx]

## Slide 1
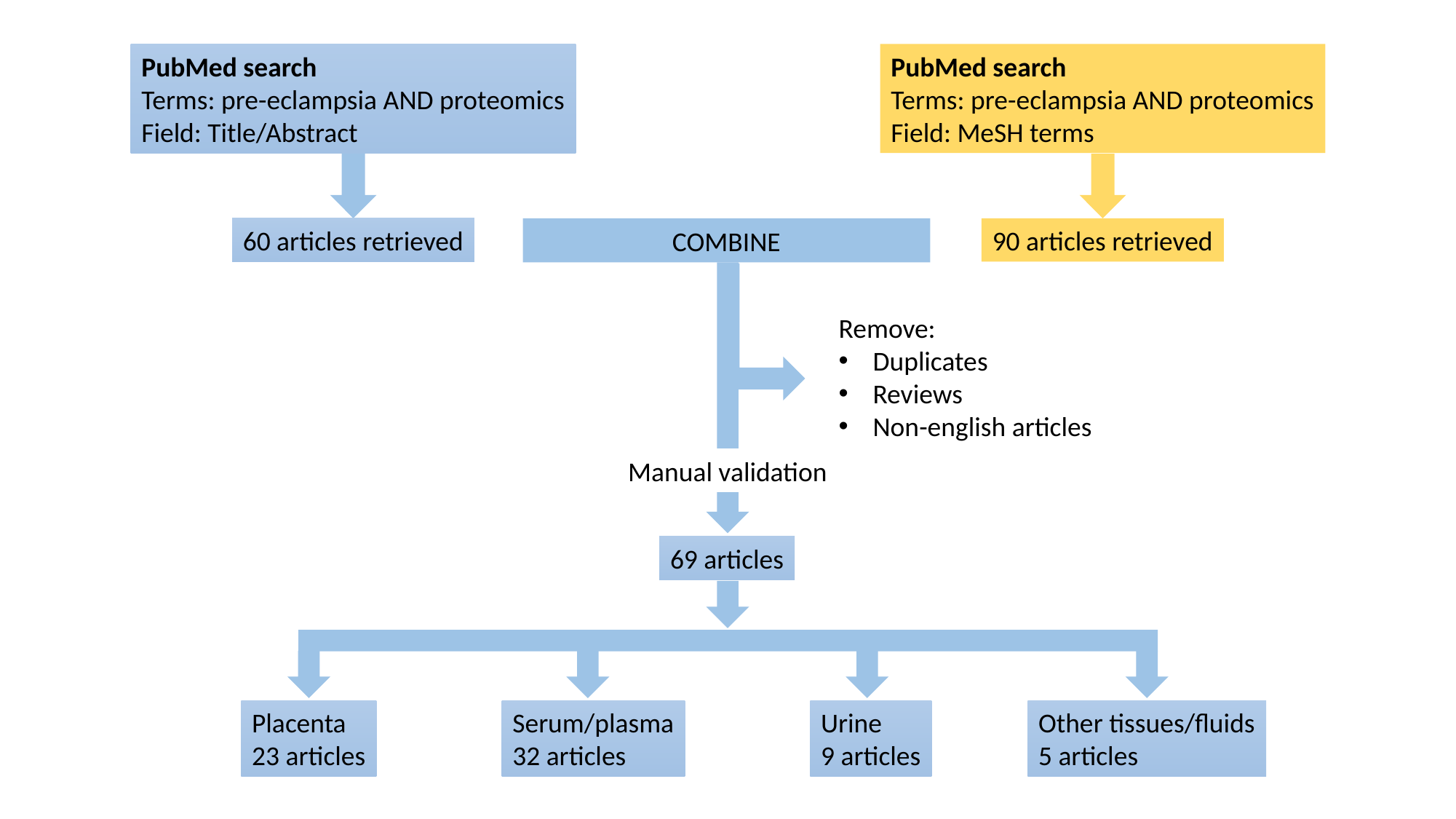

PubMed search
Terms: pre-eclampsia AND proteomics
Field: Title/Abstract
PubMed search
Terms: pre-eclampsia AND proteomics
Field: MeSH terms
60 articles retrieved
COMBINE
90 articles retrieved
Remove:
Duplicates
Reviews
Non-english articles
Manual validation
69 articles
Placenta
23 articles
Serum/plasma
32 articles
Urine
9 articles
Other tissues/fluids
5 articles
