# Supplementary material for: Quantitative proteomics-based analyses performed on pre-eclampsia samples in the 2004–2020 period: a systematic review
Source: Clin Proteomics. 2021 Jan 26;18:6. doi: 10.1186/s12014-021-09313-1 (PMC7836571; doi:10.1186/s12014-021-09313-1)
Supplement: Supplementary file 7 — Additional file 7. Supplementary methods: acceptance criteria for the proteins appearing in Tables 1, 2, 3. [file 12014_2021_9313_MOESM7_ESM.docx]

**Acceptance criteria for the proteins appearing in tables 1-3:**

For each (p), we would have:

n_p_ = x_p_+y_p_, where

n_p_ = total number of studies in which the protein is quantified

x_p_ = total number of studies in which the protein is upregulated in PE vs C (PE>C)

y_p_ = total number of studies in which the protein is downregulated in PE vs C (PE<C)

The percentage of expression pattern (% of expression pattern) is defined for each up/down regulated protein as:

% of expression pattern = x_p_*100/n_p_, for over-expression

% of expression pattern = y_p_*100/n_p,_ for under-expression

It can be assumed that the results follow a Gaussian distribution around a mean (μ) with a certain value of standard deviation (σ), where the percentage of data points that would lie within one sigma region [μ−σ, μ+σ], would be 68.3%.

The following confidence criterion is defined for each protein, leading to two groups:

- High-confidence differentially expressed protein cluster, containing those proteins with % of expression pattern > 68.3%, reported in at least 3 studies.
- Medium-confidence differentially expressed protein cluster, that would in turn comprise two categories:
  - % of expression pattern of 66.7% (slightly lower than the acceptance interval of –σ/+σ region), reported in at least 4 studies (or independent data points).
  - Consistently up/down regulated proteins included in 2 urine-based studies, as the number of publications in urine matrix is much smaller than those of serum/plasma and placenta
